# Transcriptome Profiling to Discover Putative Genes Associated with Paraquat Resistance in Goosegrass (*Eleusine indica* L.)

**DOI:** 10.1371/journal.pone.0099940

**Published:** 2014-06-13

**Authors:** Jing An, Xuefeng Shen, Qibin Ma, Cunyi Yang, Simin Liu, Yong Chen

**Affiliations:** 1 Weed Research Laboratory, College of Agriculture, South China Agricultural University, Guangzhou, Guangdong, P. R. China; 2 State Key Laboratory for Conservation and Utilization of Subtropical Agro-Bioresources, College of Agriculture, South China Agricultural University, Guangzhou, Guangdong, P. R. China; University of Hong Kong, China

## Abstract

**Background:**

Goosegrass (*Eleusine indica* L.), a serious annual weed in the world, has evolved resistance to several herbicides including paraquat, a non-selective herbicide. The mechanism of paraquat resistance in weeds is only partially understood. To further study the molecular mechanism underlying paraquat resistance in goosegrass, we performed transcriptome analysis of susceptible and resistant biotypes of goosegrass with or without paraquat treatment.

**Results:**

The RNA-seq libraries generated 194,716,560 valid reads with an average length of 91.29 bp. *De novo* assembly analysis produced 158,461 transcripts with an average length of 1153.74 bp and 100,742 unigenes with an average length of 712.79 bp. Among these, 25,926 unigenes were assigned to 65 GO terms that contained three main categories. A total of 13,809 unigenes with 1,208 enzyme commission numbers were assigned to 314 predicted KEGG metabolic pathways, and 12,719 unigenes were categorized into 25 KOG classifications. Furthermore, our results revealed that 53 genes related to reactive oxygen species scavenging, 10 genes related to polyamines and 18 genes related to transport were differentially expressed in paraquat treatment experiments. The genes related to polyamines and transport are likely potential candidate genes that could be further investigated to confirm their roles in paraquat resistance of goosegrass.

**Conclusion:**

This is the first large-scale transcriptome sequencing of *E. indica* using the Illumina platform. Potential genes involved in paraquat resistance were identified from the assembled sequences. The transcriptome data may serve as a reference for further analysis of gene expression and functional genomics studies, and will facilitate the study of paraquat resistance at the molecular level in goosegrass.

## Introduction

*Eleusine indica* L. (Gaertn), commonly known as goosegrass, is a monocot weed belonging to the Poaceae family [1]. Due to its high fecundity and a wide tolerance to various environmental factors, goosegrass is listed as one of the five most noxious weeds in the world and has been reported to be a problem weed for 46 different crop species in more than 60 countries [1]. Many herbicides are being used to control goosegrass, i.e., bipyridinium herbicides such as N, N′-dimethyl-4, 4′-bipyridinium dichloride (paraquat); dinitroaniline herbicides; acetohydroxyacid synthase inhibitors such as imazapyr; and acetyl CoA carboxylase inhibitors such as fluazifop, glyphosate and glufosinate. However, application of the same herbicide for more than three consecutive years resulted in goosegrass populations that acquired resistance to the herbicide [2]–[7]. Paraquat, a quick-acting herbicide widely used for the non-selective control of weeds both in field crops and orchards, causes plant mortality by diverting electrons from photosystem I to molecular oxygen, resulting in a serious oxidative damage to the exposed tissues [8]–[9]. Weeds can acquire resistance to paraquat from extensive exposure (over a period of >10 years) to the herbicide [10]–[12].

Current understanding of the molecular mechanism of paraquat resistance in higher plants includes sequestration of paraquat to the vacuoles and/or enhanced activity of antioxidative enzymes [13]–[14]. Putrescine has been reported as a competitive inhibitor of energy-dependent, saturable transporters that facilitate paraquat transport across the plasma membrane [15]–[17], suggesting resistance to paraquat can likely be improved by modulating the activity of its transporters [9]. Further characterization of resistance mechanisms evolved in *E. indica* and other weeds to paraquat has been hindered due to the lack of genome-level information in these species. Next-generation sequencing (NGS) technology has rapidly advanced the analysis of genomes and transcriptomes in model plant and crop species which can now be applied to other species whose genomes have not been sequenced [18]–[19]. NGS has also been widely used for comparative transcriptome analysis to identify genes that are differentially expressed across different cultivars or tissues or treatment conditions [20]–[23].

**Figure 1:**
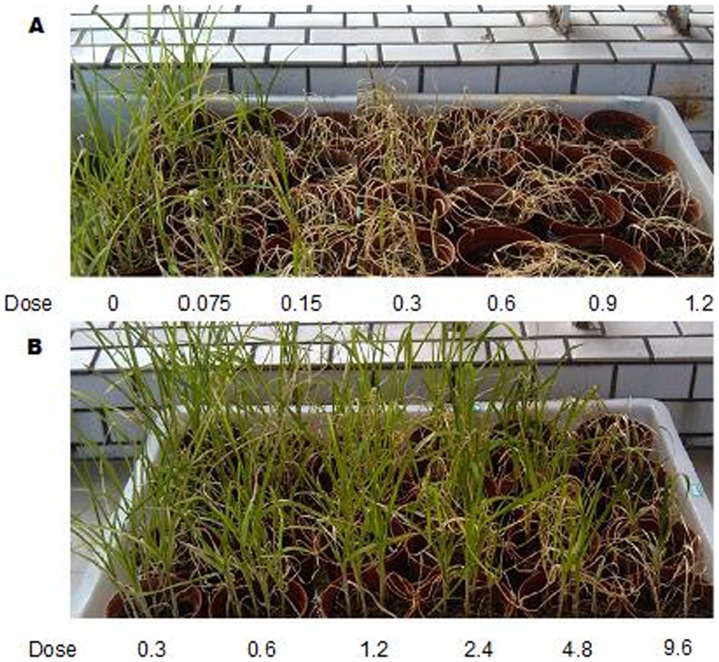
The growth of susceptible (A) and resistant (B) goosegrass biotype at various concentrations of paraquat. The dose of paraquat is kg·ai·ha^−1^.

In this study, we explored the paraquat resistance mechanisms in resistant and susceptible biotypes of *E. indica* (Figure 1) by generating comprehensive *de novo* transcriptome datasets using Illumina platform. Analysis of the gene expression data identified unigenes that were assigned to various GO categories and KEGG metabolic pathways which can be used for further molecular characterization of paraquat resistance mechanisms in *E. indica*.

## Results

### Illumina Sequencing and *de novo* Assembly

**Figure 2:**
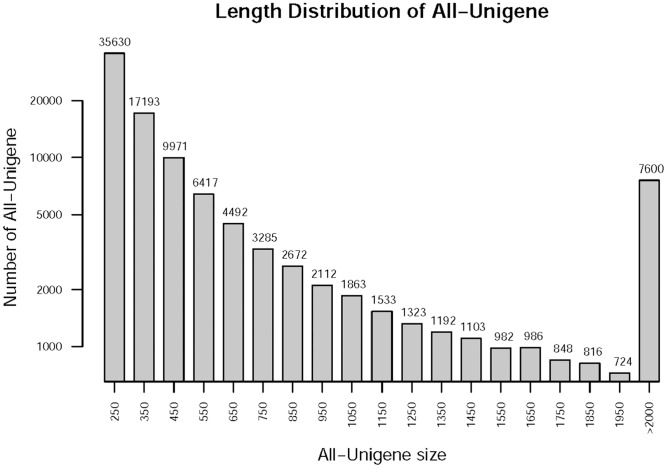
Length distribution of unigenes characterized from RNA-seq libraries of goosegrass.

Four RNA-seq libraries sequenced from goosegrass seedlings were named based on their respective samples: S0 - susceptible seedlings without paraquat; SQ - susceptible seedlings for mixed samples sprayed paraquat 40 min, 60 min and 80 min; R0 - resistant seedlings without paraquat; and RQ - resistant seedlings for mixed samples sprayed paraquat 40 min, 60 min and 80 min. S0, SQ, R0 and RQ libraries generated 57.25, 61.44, 66.51 and 58.66 million raw reads, respectively (Table 1). More than 79.85% of all the raw reads used for *de novo* assembly had Phred-like quality scores at the Q20 level (an error probability of 1%). We obtained 158,461 (>200 bp) transcripts with an average length of 1,153.74 bp and an N50 of 2,095 bp. 100,742 (>200 bp) unigenes with an average length of 712.79 bp and an N50 of 1,199 bp were obtained by using longest transcript in each loci as unigene (Table 2). The statistical results showed reducing trend of unigene number with increasing length of unigenes. Sequence length distribution of unigenes changed from 250 bp to 2000 bp (Figure 2).

**Table 1:** Summary of goosegrass transcriptome sequencing.

Sample	Raw Data		Valid Data			Valid Ratio (reads)
	Read	Base	Read	Base	Average length	
S0	57,251,668	5,725,166,800	45,250,056	4,130,568,660	91.28	79.04%
SQ	61,444,178	6,144,417,800	50,266,462	4,621,243,200	91.93	81.81%
R0	66,507,532	6,650,753,200	52,712,154	4,792,947,566	90.93	79.26%
RQ	58,663,262	5,866,326,200	46,487,888	4,231,153,734	91.02	79.25%
All	243,866,640	24,386,664,000	194,716,560	17,775,913,160	91.29	79.85%

S0: susceptible goosegrass seedlings without paraquat; SQ: susceptible goosegrass seedlings for mixed samples sprayed paraquat 40 min, 60 min and 80 min; R0: resistant goosegrass seedlings without paraquat; RQ: resistant goosegrass seedlings for mixed samples sprayed paraquat 40 min, 60 min and 80 min.

**Table 2:** *De novo* assembly results of goosegrass transcriptome.

	All (≥200 bp)	≥500 bp	≥1000 bp	N50	N90	Total Length	Max Length	Min Length	Average Length
Transcript	158,461	90,029	61,339	2,095	445	182,822,211	16,925	2,01	1153.74
Unigene	100,742	38,049	18,993	1,199	280	71,807,744	16,925	2,01	712.79

N50: 50% of the assembled bases were incorporated into sequences with length of N50 or longer. N90: 90% of the assembled bases were incorporated into sequences with length of N90 or longer.

### Functional annotation of assembled unigenes

**Figure 3:**
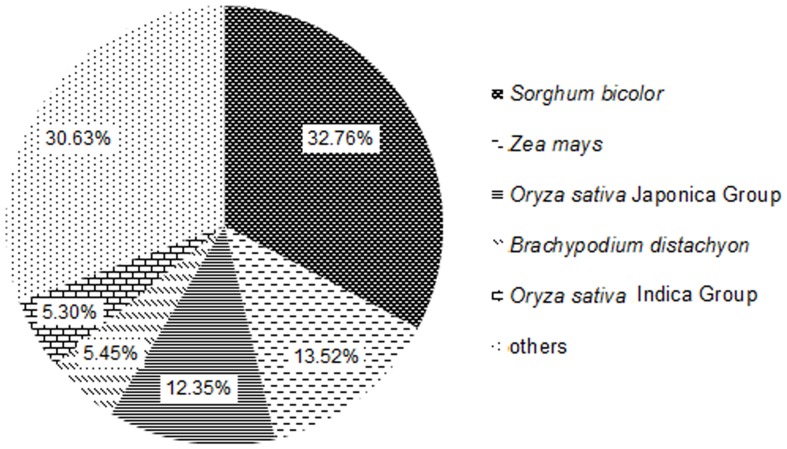
Percentage of conservation of goosegrass unigenes in different monocot species based on top BLAST hits.

To study the sequence conservation of goosegrass genes with other plant species, we used an E-value threshold of 10^−5^ to annotate 35,016 (34.76%), 19,921 (19.77%), 35,983 (35.72%), 17,574 (17.44%), 31,584 (31.35%) and 12,719 (12.63%) unigenes to nr [24], Swiss-Prot [25], TrEMBL [26], CDD [27], Pfam [28] and KOG [29] databases, respectively. The BLAST [30] results of sequences indicated that 35,016 unigenes had BLAST hits in nucleotide sequence database in NCBI database. The majority of the annotated nucleotide sequences of goosegrass corresponded to those of Poaceae plant species, which including *Sorghum bicolor*, *Zea mays*, *Oryza sativa* Japonica Group, *Brachypodium distachyon* and *Oryza sativa* Indica Group with matching ratios of 32.76%, 13.52%, 12.35%, 5.45% and 5.30%, respectively (Figure 3).

**Figure 4:**
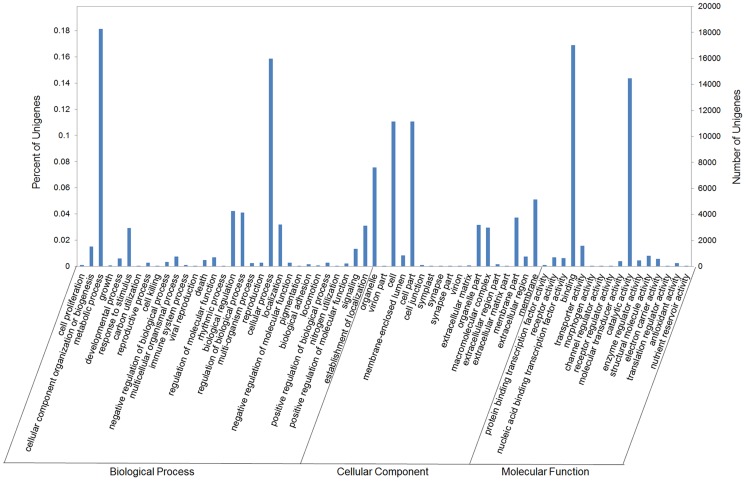
GO classifications of goosegrass unigenes. The results were summarized in three main categories: biological process, cellular component and molecular function by GO analysis.

Gene ontology assignments were used to classify the functions of goosegrass transcripts. A total of 25,926 unigenes (25.74%) were assigned at least one GO term and classified into 65 functional categories using the complete set of GO terms for three main categories: biological process, cellular component and molecular function (Figure 4). The largest proportion was represented by metabolic process (GO: 0008152, 18.13%) and cellular process (GO: 0009987, 15.87%) under biological process; cell (GO: 0005623, 11.06%) and cell part (GO: 0044464, 11.06%) under cellular component; binding (GO: 0005488, 16.88%) and catalytic activity (GO: 0003824, 14.37%) under molecular function.

**Figure 5:**
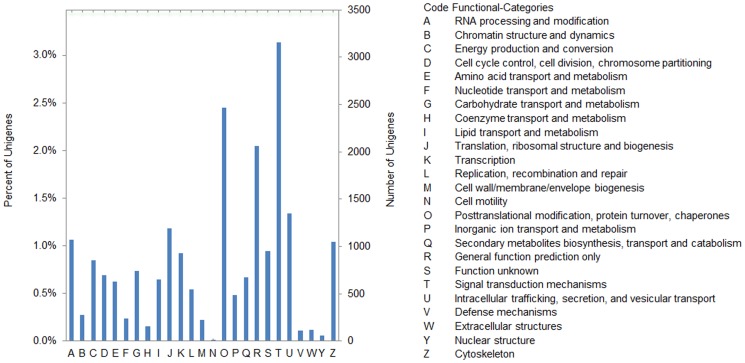
KOG classification of putative proteins corresponding to goosegrass unigenes. All 12,719 putative proteins shown significant homology to those in KOG database were function classified into 25 molecular families. Right Y-axis indicates percentage of a specific category of genes in each main classification. Left Y-axis represents number of genes in a classification.

In total, 12,719 unigenes were categorized into 25 KOG classifications (Figure 5). Among these categories, the cluster for “signal transduction mechanisms” (3,160, 24.84%) was the largest group, followed by the categories of “posttranslational modification, protein turnover, chaperones” (2,464, 19.37%), “general function prediction only” (2,062, 16.21%), “intracellular trafficking, secretion and vesicular transport” (1,352, 10.63%) and “translation, ribosomal structure and biogenesis” (1,195, 9.40%). The categories of “cell motility” (17, 0.13%) and “nuclear structure” (60, 0.47%) had the fewest corresponding genes.

The 100,742 assembled sequences were mapped to the reference canonical pathways in KEGG. A total of 13,809 unigenes with 1,208 enzyme commission (EC) numbers were assigned to 314 predicted KEGG metabolic pathways. The pathways most strongly represented by mapped unigenes were “ribosome” (ko 03010, 523 unigenes), “protein processing in endoplasmic reticulum” (ko 04141, 415 unigenes), “spliceosome” (ko 03040, 408 unigenes), “RNA transport” (ko 03013, 345 unigenes) and “plant-pathogen interaction” (ko 04626, 326 unigenes).

### Identification and annotation of differentially expressed genes (DEGs)

**Figure 6:**
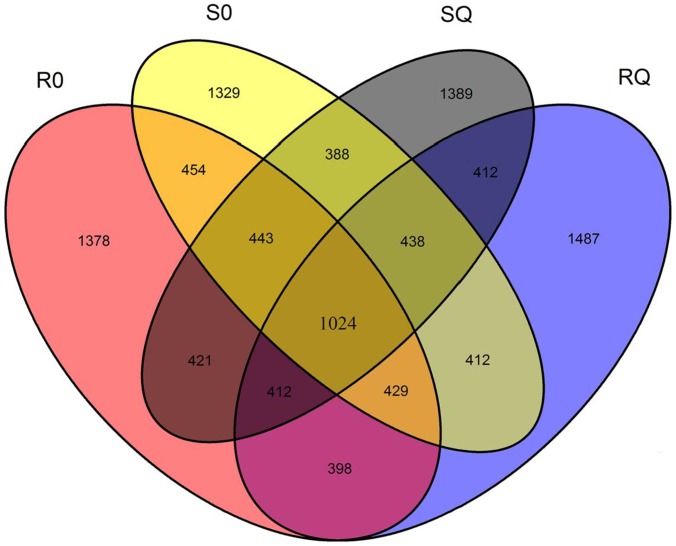
Venn diagram showing the genes expressed in each of four samples of goosegrass transcriptomes (RPKM>10). S0: susceptible goosegrass seedlings without paraquat; SQ: susceptible goosegrass seedlings for mixed samples sprayed paraquat 40 min, 60 min and 80 min; R0: resistant goosegrass seedlings without paraquat; RQ: resistant goosegrass seedlings for mixed samples sprayed paraquat 40 min, 60 min and 80 min.

Transcripts expression levels were calculated using RPKM (Reads per kilobase of exon model per million mapped reads). The expression differences of transcripts among the four samples of S0, SQ, R0 and RQ are summarized in a venn diagram that clearly showed the overlapping relationship (Figure 6). Among all the transcripts (RPKM>10), 1,024 transcripts were expressed at all of the four samples, 388, 398, 454 and 412 transcripts were co-expressed in treatments of S0 and SQ, R0 and RQ, S0 and R0, SQ and RQ, respectively. The numbers of each sample specifically expressed transcripts was 1,329 (S0), 1,389 (SQ), 1,378 (R0) and 1,487 (RQ), respectively.

**Figure 7:**
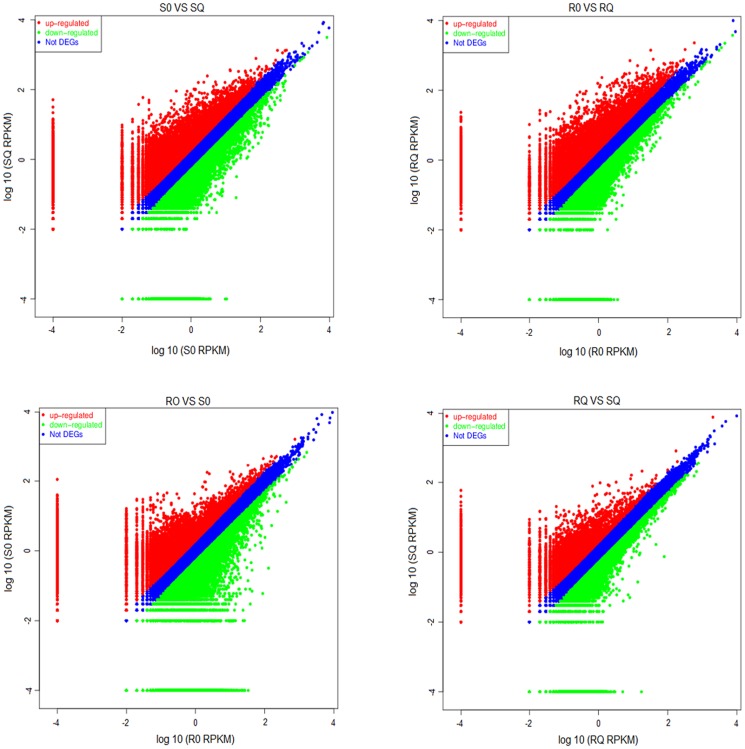
Scatter plot analysis of four sample pairs (S0 vs SQ, R0 vs RQ, R0 vs S0 and RQ vs SQ) from goosegrass. DEGs were determined using a threshold of log_2_ Ratio ≥1 and FDR≤0.001. S0: susceptible goosegrass seedlings without paraquat; SQ: susceptible goosegrass seedlings for mixed samples sprayed paraquat 40 min, 60 min and 80 min; R0: resistant goosegrass seedlings without paraquat; RQ: resistant goosegrass seedlings for mixed samples sprayed paraquat 40 min, 60 min and 80 min. Red spots represent up-regulated DEGs and green spots indicate down-regulated DEGs. Those shown in blue are Transcripts that did not show obvious changes.

In differentially expressed genes (DEG) analysis, we defined DEG as the fold change of the normalized (RPKM) expression values of at least 2 in both directions of log_2_ ratio≥1 and false discovery rate (FDR)≤0.001 (Figure 7). In total, 35,569 DEGs were up-regulated and 32,500 DEGs were down-regulated between the samples S0 and SQ; 30,518 DEGs were up-regulated and 35,020 DEGs were down-regulated between the samples of R0 and RQ; 34,579 DEGs were up-regulated and 32,515 DEGs were down-regulated between the samples of R0 and S0; and 33,722 DEGs were up-regulated and 22,902 DEGs were down-regulated between the samples of RQ and SQ.

### Comparison of transcripts involved in paraquat resistance

#### ROS pathway

Many genes related to reactive oxygen species (ROS) removal pathway were differentially regulated in goosegrass biotypes after paraquat treatment which likely contributes to their susceptibility or resistance to paraquat. Of the 53 identified genes that function in the ROS scavenging pathway: 11 belonged to the glutathione-ascorbate cycle (glutaredoxin, GLR; monodehydroascorbate reductase, MDAR; and glutathione reductase, GR); 34 to the glutathione peroxidase (glutathione, GST; and peroxidases, POD); 5 to the catalase (CAT) pathway; and 3 to the thioredoxin (Trx) pathway (Table 3). The three largest groups of ROS related genes were GST (24 genes), POD (10 genes) and GLR (7 genes). Highest transcript levels were observed for three genes in all the four samples, i.e., POD (comp 31277_c0_seq3), CAT (comp 34816_c0_seq1) and Trx (comp 34820_c0_seq1). DEGs analysis revealed that most of ROS pathway genes are up-regulated both in resistant and susceptible biotypes of *E. indica* after application of paraquat (Table 3). DEGs in the ROS pathway that were down-regulated in treatment comparisons are as follows: SQ vs S0 - two GLR genes (comp 38421_c0_seq1 and comp 23286_c0_seq1) and one POD (comp 14213_c0_seq1); RQ vs R0 - two GLR genes (comp 38421_c0_seq1 and comp 23286_c0_seq1), one GR (comp 41205_c0_seq1), two GST (comp 38536_c0_seq1 and comp 8718_c0_seq1), three POD (comp 41522_c0_seq1, comp 29849_c0_seq2 and comp 14213_c0_seq1) and one CAT (comp 34816_c0_seq1). However, R0 vs S0 comparison revealed: up-regulation (>2-fold) of one GST (comp 38536_c0_seq1) and two POD (comp 41522_c0_seq1 and comp 13511_c0_seq1); and down-regulation of one GLR (comp 23286_c0_seq1) and five GST (comp 29100_c0_seq3, comp 27832_c0_seq2, comp 16427_c0_seq2, comp 31694_c0_seq1 and comp 32752_c0_seq3). Whereas in RQ vs SQ comparison, one MDAR (comp 29012_c0_seq4) and one GST (comp 17835_c0_seq1) was up-regulated (>1-fold), one GLR (comp 38421_c0_seq1) and one POD (comp 29849_c0_seq2) were down-regulated (>1-fold) (Table 3).

**Table 3:** DEGs and highly expressed goosegrass transcripts related to ROS scavenging system.

Gene ID	RPKM				Fold change				Homologous species
	S0	SQ	R0	RQ	SQ/S0	RQ/R0	R0/S0	RQ/SQ	
Glutaredoxin, GLR									
comp15376_c0_seq1	1.29	14.44	2.24	10.85	+3.48	+2.28	+0.80	−0.41	*Brachypodium distachyon*
comp26674_c0_seq1	5.33	25.47	4.43	31.31	+2.26	+2.82	−0.27	+0.30	*Sorghum bicolor*
comp31422_c0_seq2	17.04	54.86	6.61	64.96	+1.69	+3.30	−1.37	+0.24	*Sorghum bicolor*
comp18533_c0_seq1	7.88	17.38	6.96	21.07	+1.14	+1.60	−0.18	+0.28	*Sorghum bicolor*
comp12002_c0_seq1	9.64	13.29	4.95	14.27	+0.46	+1.53	−0.96	+0.10	*Brachypodium distachyon*
comp38421_c0_seq1	11.81	11.68	17.76	5.66	−0.02	−1.65	+0.59	−1.05	*Sorghum bicolor*
comp23286_c0_seq1	12.73	1.05	2.49	2.09	−3.60	−0.25	−2.35	+0.99	*Zea mays*
Monodehydroascorbate reductase, MDAR									
comp29012_c0_seq4	8.72	87.61	3.10	182.07	+3.33	+5.88	−1.49	+1.06	*Zea mays*
Glutathione reductase, GR									
comp34945_c0_seq1	98.16	285.68	95.74	391.53	+1.54	+2.03	−0.04	+0.45	*Oryza sativa* Japonica Group
comp31724_c0_seq5	3.35	7.08	2.96	10.39	+1.08	+1.81	−0.18	+0.55	*Oryza sativa* Japonica Group
comp41205_c0_seq1	4.02	7.00	12.02	9.85	+0.80	−0.29	+1.58	+0.49	*Oryza sativa* Japonica Group
Glutathione S-transferase, GST									
comp15320_c0_seq2	0.02	11.99	0.02	21.09	+9.23	+10.04	0	+0.81	*Zea mays*
comp26759_c0_seq1	0.04	12.94	0	23.28	+8.34			+0.85	*Zea mays*
comp29100_c0_seq3	0.19	16.58	0.03	19.26	+6.45	+9.33	−2.66	+0.22	*Hordeum vulgare* subsp. vulgare
comp18690_c0_seq1	1.47	30.41	0.91	52.89	+4.37	+5.86	−0.69	+0.80	*Sorghum bicolor*
comp26545_c0_seq1	23.36	331.94	13.5	507.51	+3.83	+5.23	−0.79	+0.61	*Sorghum bicolor*
comp35892_c0_seq1	7.41	83.19	5.11	100.99	+3.49	+4.30	−0.54	+0.28	*Sorghum bicolor*
comp37758_c0_seq1	4.68	42.10	13.58	21.56	+3.17	+0.67	+1.54	−0.97	*Hordeum vulgare* subsp. vulgare
comp19673_c0_seq1	39.56	255.53	18.43	270.78	+2.69	+3.88	−1.10	+0.08	*Triticum aestivum*
comp29023_c0_seq11	2.54	15.43	1.11	24.72	+2.60	+4.48	−1.19	+0.68	*Zea mays*
comp38536_c0_seq1	1.65	9.75	23.19	8.83	+2.56	−1.39	+3.81	−0.14	*Oryza sativa* Japonica Group
comp27832_c0_seq2	3.77	20.63	0.27	29.32	+2.45	+6.76	−3.80	+0.51	*Sorghum bicolor*
comp16427_c0_seq2	7.54	38.32	0.15	31.91	+2.35	+7.73	−5.65	−0.26	*Oryza sativa* Japonica Group
comp30904_c0_seq5	5.97	26.37	1.89	20.79	+2.14	+3.46	−1.66	−0.34	*Brachypodium distachyon*
comp17835_c0_seq1	10.42	42.08	11.14	88.12	+2.01	+2.98	+0.10	+1.07	*Sorghum bicolor*
comp28455_c0_seq1	4.39	15.96	7.33	8.27	+1.86	+0.17	+0.74	−0.95	*Oryza sativa* Japonica Group
comp35531_c0_seq1	23.26	83.60	42.07	57.04	+1.85	+0.44	+0.85	−0.55	*Zea mays*
comp31694_c0_seq1	42.86	123.24	0.29	88.89	+1.52	+8.26	−7.21	−0.47	*Sorghum bicolor*
comp32752_c0_seq3	4.82	13.80	0.88	27.31	+1.52	+4.96	−2.45	+0.98	*Sorghum bicolor*
comp8070_c0_seq1	14.00	36.37	30.64	40.04	+1.38	+0.39	+1.13	+0.14	*Hordeum vulgare* subsp. vulgare
comp8717_c0_seq1	14.41	37.40	24.8	22.02	+1.38	−0.17	+0.78	−0.76	*Zea mays*
comp20535_c0_seq3	29.25	69.99	10.51	113.38	+1.26	+3.43	−1.48	+0.70	*Zea mays*
comp26298_c0_seq1	16.71	37.74	9.97	66.50	+1.18	+2.74	−0.75	+0.82	*Zea mays*
comp29106_c0_seq1	43.94	92.17	57.11	147.96	+1.07	+1.37	+0.38	+0.68	*Oryza sativa* Japonica Group
comp30519_c0_seq2	33.13	50.58	22.87	50.24	+0.61	+1.14	−0.53	−0.01	*Cynodon dactylon*
Peroxidases, POD									
comp23196_c0_seq1	1.81	23.47	1.34	19.85	+3.70	+3.89	−0.43	−0.24	*Hordeum vulgare* subsp. vulgare
comp41522_c0_seq1	1.02	12.56	14.42	8.91	+3.62	−0.69	+3.82	−0.50	*Oryza sativa* Japonica Group
comp33361_c1_seq2	6.84	52.06	15.37	70.43	+2.93	+2.20	+1.17	+0.44	*Sorghum bicolor*
comp13511_c0_seq1	8.94	53.01	50.19	67.70	+2.57	+0.43	+2.49	+0.35	*Sorghum bicolor*
comp29849_c0_seq2	5.16	23.55	12.51	7.88	+2.19	−0.67	+1.28	−1.58	*Sorghum bicolor*
comp36428_c0_seq1	15.10	64.73	24.98	85.71	+2.10	+1.78	+0.73	+0.41	*Eleusine coracana*
comp6539_c0_seq1	17.12	64.03	42.95	59.89	+1.90	+0.48	+1.33	−0.10	*Oryza sativa* Japonica Group
comp33652_c1_seq12	3.69	9.26	1.75	14.01	+1.33	+3.00	−1.08	+0.60	*Sorghum bicolor*
comp31277_c0_seq3	254.28	507.65	174.07	487.62	+1.00	+1.49	−0.55	−0.06	*Sorghum bicolor*
comp14213_c0_seq1	61.08	2.22	23.54	3.10	−4.78	−2.92	−1.38	+0.48	*Zea mays*
Catalase, CAT									
comp40218_c0_seq1	6.27	16.80	14.15	32.85	+1.42	+1.22	+1.17	+0.97	*Brachypodium distachyon*
comp8916_c0_seq1	67.39	134.66	115.3	161.87	+1.00	+0.49	+0.77	+0.27	*Zea mays*
comp37657_c0_seq1	17.67	29.26	18.88	48.77	+0.73	+1.37	+0.10	+0.74	*Brachypodium distachyon*
comp34816_c0_seq1	695.16	1073.47	1654.64	644.10	+0.63	−1.36	+1.25	−0.74	*Zea mays*
comp35271_c0_seq1	90.11	102.77	60.06	129.28	+0.19	+1.11	−0.59	+0.33	*Oryza sativa* Japonica Group
Thioredoxin, Trx									
comp34820_c0_seq1	438.31	1133.65	319.84	1474.23	+1.37	+2.20	−0.45	+0.38	*Sorghum bicolor*
comp24761_c0_seq1	48.66	71.98	37.84	85.03	+0.56	+1.17	−0.36	+0.24	*Zea mays*
comp5507_c0_seq1	13.50	15.51	8.11	16.28	+0.20	+1.01	−0.74	+0.07	*Helianthus annuus*

DEGs and highly transcribed Transcripts was filtered using at least one RPKM values of Transcripts ≥ 10 among S0, SQ, R0 and RQ; and at least one absolute value of Fold Change ≥1 among SQ/S0, RQ/R0, R0/S0 and RQ/SQ. “RPKM” indicates RPKM values of Transcripts in S0, SQ, R0 and RQ. “Fold Change” equals to log _2_ (A/B). “+” indicates up-regulated transcription and “-” represents down-regulated transcription. Homologous species is that identified from BLAST search of nr database using the cut-off E-value of ≤10^−5^.

#### Polyamine metabolism

With reference to genes that are related to polyamine metabolism, 10 DEGs were found to encode enzymes that catalyze polyamine turnover (Table 4). In two comparisons of SQ vs S0 and R0 vs S0, two genes (comp 30623_c0_seq2 and comp 4323_c0_seq1) were down-regulated whereas others were all up-regulated. Only two genes (comp 30798_c0_seq2 and comp 10199_c0_seq1) were up-regulated between RQ and R0; comp 30798_c0_seq2 was up-regulated with 1.07 fold change between RQ and SQ, while the expressions of other genes were not significantly altered (fold change ≤1) (Table 4).

**Table 4:** DEGs and highly expressed goosegrass transcripts related to polyamines.

Gene ID	RPKM				Fold change				Homologous species
	S0	SQ	R0	RQ	SQ/S0	RQ/R0	R0/S0	RQ/SQ	
comp28690_c0_seq2	4.87	18.28	10.77	10.27	+1.91	−0.07	+1.15	−0.83	*Sorghum bicolor*
comp30798_c0_seq2	21.44	74.81	59.88	156.83	+1.80	+1.39	+1.48	+1.07	*Zea mays*
comp10199_c0_seq1	5.11	11.04	7.91	14.46	+1.11	+0.87	+0.63	+0.39	*Zea mays*
comp13648_c0_seq1	8.16	16.88	19.30	12.09	+1.05	−0.67	+1.24	−0.48	*Zea mays*
comp19110_c0_seq1	4.79	9.23	12.14	9.67	+0.95	−0.33	+1.34	+0.07	*Hordeum vulgare* subsp. vulgare
comp25370_c0_seq2	11.45	18.48	26.21	19.64	+0.69	−0.42	+1.19	+0.09	*Sorghum bicolor*
comp3931_c0_seq1	2.20	2.76	12.92	2.36	+0.33	−2.45	+2.55	−0.23	*Sorghum bicolor*
comp36158_c0_seq1	24.81	26.74	52.60	28.79	+0.11	−0.87	+1.08	+0.11	*Sorghum bicolor*
comp30623_c0_seq2	20.52	9.79	13.47	10.02	−1.07	−0.43	−0.61	+0.03	*Zea mays*
comp4323_c0_seq1	11.30	3.14	5.45	4.78	−1.85	−0.19	−1.05	+0.61	*Sorghum bicolor*

DEGs and highly transcribed Transcripts was filtered using at least one RPKM values of Transcripts ≥10 among S0, SQ, R0 and RQ; and at least one absolute value of Fold Change ≥1 among SQ/S0, RQ/R0, R0/S0 and RQ/SQ. “RPKM” indicates RPKM values of Transcripts in S0, SQ, R0 and RQ. “Fold Change” equals to log _2_ (A/B). “+” indicates up-regulated transcription and “-” represents down-regulated transcription. Homologous species is that identified from BLAST search of nr database using the cut-off E-value of ≤10^−5^.

#### Transporter related genes

Among the transcripts related to transmembrane transport, intracellular protein transport and ATP binding cassette transporters (ABC transporters), we identified 9, 5 and 4 genes, respectively (Table 5). Between SQ and S0, only two transmembrane transport genes (comp 28899_c0_seq1 and comp 28988_c1_seq1) and one ABC transporters gene (comp 18030_c0_seq1) were down-regulated, other 15 genes were up-regulated. In comparison of RQ vs R0, 11 genes were up-regulated, while two ABC transporters genes of comp 34549_c0_seq1 and comp 23747_c0_seq2 were up-regulated (>2-fold). 7 genes were down-regulated, while two genes of comp 10856_c0_seq1 (transmembrane transport gene) and comp 30571_c0_seq1 (ABC transporters gene) were down-regulated (>1.3 fold). Between R0 and S0, 11 genes were up-regulated. The greatest up-regulated genes were comp 10856_c0_se1 (transmembrane transport gene) (>2.44 fold) and comp 37714_c0_seq1 (intracellular protein transport gene) (>2.98 fold). Two ABC transporters genes (comp 34549_c0_seq1 [−1.06 fold] and comp 18030_c0_seq1 [−1.32 fold]) were maximally down-regulated among the 7 down-regulated genes. In RQ vs SQ comparison, only three genes, one transmembrane transport gene (comp 37518_c0_seq1 [−0.05 fold]), two intracellular protein transport genes (comp 25903_c0_seq1 [−0.35 fold] and comp 9251_c0_seq1 [−0.13 fold]) were down-regulated. 5 genes that showed highest expression among the 15 up-regulated genes included: one transmembrane transport gene (comp 9121_c0_seq1 [1.03 fold]); two intracellular protein transporters, (comp 37714_c0_seq1 [1.26 fold]) and (comp 34970_c0_seq1 [1.01 fold]); and two ABC transporters genes, (comp 34549_c0_seq1 [1.03 fold]) and (comp 23747_c0_seq2 [1.20 fold]).

**Table 5:** DEGs and highly expressed goosegrass transcripts related to transport.

Gene ID	RPKM				Fold change				Homologous species
	S0	SQ	R0	RQ	SQ/S0	RQ/R0	R0/S0	RQ/SQ	
Transmembrane transport									
comp9121_c0_seq1	24.85	53.23	33.89	108.93	+1.10	+1.68	+0.45	+1.03	*Sorghum bicolor*
comp37518_c0_seq1	13.68	28.70	15.76	27.69	+1.07	+0.81	+0.20	−0.05	*Zea mays*
comp26346_c0_seq2	4.41	7.49	4.60	12.39	+0.76	+1.43	+0.06	+0.73	*Sorghum bicolor*
comp27044_c1_seq1	11.68	19.72	24.66	30.43	+0.76	+0.30	+1.08	+0.63	*Oryza sativa* Indica Group
comp16638_c0_seq1	20.09	27.91	41.22	46.67	+0.47	+0.18	+1.04	+0.74	*Oryza sativa* Japonica Group
comp10856_c0_seq1	9.83	12.16	53.19	18.53	+0.31	−1.52	+2.44	+0.61	*Zea mays*
comp29259_c0_seq1	11.59	11.9	26.14	21.11	+0.04	−0.31	+1.17	+0.83	*Sorghum bicolor*
comp28899_c0_seq1	11.60	5.53	7.87	6.18	−1.07	−0.35	−0.56	+0.16	*Hordeum vulgare* subsp. vulgare
comp28988_c1_seq1	102.77	47.81	94.06	59.38	−1.10	−0.66	−0.13	+0.31	*Aeluropus lagopoides*
Iintracellular protein transport									
comp25903_c0_seq1	26.61	69.45	26.14	54.33	+1.38	+1.06	−0.03	−0.35	*Festuca arundinacea*
comp37714_c0_seq1	3.45	8.76	27.14	20.91	+1.34	−0.38	+2.98	+1.26	*Sorghum bicolor*
comp34970_c0_seq1	46.18	88.18	97.54	177.02	+0.93	+0.86	+1.08	+1.01	*Sorghum bicolor*
comp9251_c0_seq1	52.15	83.68	37.40	76.25	+0.68	+1.03	−0.48	−0.13	*Zea mays*
comp35460_c0_seq1	26.14	41.12	58.00	45.21	+0.65	−0.36	+1.15	+0.14	*Sorghum bicolor*
ABC transporters									
comp34549_c0_seq1	2.11	23.99	1.01	49.13	+3.51	+5.60	−1.06	+1.03	*Oryza sativa* Indica Group
comp23747_c0_seq2	11.52	19.87	9.25	45.80	+0.79	+2.31	−0.32	+1.20	*Brachypodium distachyon*
comp30571_c0_seq1	2.99	3.67	11.72	4.67	+0.30	−1.33	+1.97	+0.35	*Sorghum bicolor*
comp18030_c0_seq1	15.95	11.21	6.38	14.22	−0.51	+1.16	−1.32	+0.34	*Sorghum bicolor*

DEGs and highly transcribed Transcripts was filtered using at least one RPKM values of Transcripts ≥10 among S0, SQ, R0 and RQ; and at least one absolute value of Fold Change ≥1 among SQ/S0, RQ/R0, R0/S0 and RQ/SQ. “RPKM” indicates RPKM values of Transcripts in S0, SQ, R0 and RQ. “Fold Change” equals to log _2_ (A/B). “+” indicates up-regulated transcription and “-” represents down-regulated transcription. Homologous species is that identified from BLAST search of nr database using the cut-off E-value of ≤10^−5^.

## Discussion

### Construction of the transcriptome dataset for *E. indica*

For many non-model species, there is very little genome information available for researchers to conduct comprehensive investigations into the genetic mechanisms underlying their unique features and functions. The recent advances in next-generation sequencing technology has been used widely to explore genome and transcriptome information associated with important physiological phenomena in many plant species [21]. Our study has generated the first large-scale transcriptome data for goosegrass herbicide resistance using high-throughput Illumina sequencing. Comparison of the susceptible biotype with the paraquat-resistant biotype of goosegrass revealed gene expression regulation network that will be helpful to understand the molecular, biochemical and physiological processes underlying the paraquat resistance mechanism in goosegrass.

When paraquat is applied to plants, it causes rapid scorching of green tissue following exposure to light, typically within 30 min [31]. Therefore, the aerial parts of goosegrass seedlings from the two lines and treatments were used to construct RNA-seq libraries to perform comparative analysis of DEGs that will likely reveal the mechanism of paraquat resistance. To ensure that the mRNAs used for RNA-seq was the available but not-degradable RNA, we mixed the samples from the equivalent seedlings sprayed paraquat 40 min, 60 min and 80 min [32].

Our analysis of RNA-seq data (194,716,560 sequence reads categorized into 158,461 assembled transcripts) identified 100,742 unigenes, which is significantly larger than those previously reported for several transcriptomes analyzed for abiotic stress responses (e.g. 29,056 [23], 60,765 [20], 65,340 [21], 79,082 [22]). 35,016 unigenes were annotated by nr database from 100,742 unigenes. Although a high number of unigenes were not covered the complete protein-coding regions as revealed by BLAST alignment, the dataset we reported here still provided the largest dataset of different genes representing a substantial part of the transcriptome of goosegrass, which probably embraces the majority part of genes involved in the sophisticated regulation networks for resistant paraquat. The top five species with BLAST hits to annotated unigenes from goosegrass were *Sorghum bicolor*, *Zea mays*, *Oryza sativa* Japonica Group, *Brachypodium distachyon* and *Oryza sativa* Indica Group indicative of the conserved genes across monocot plant species.

### Genes involved in paraquat resistance

It is well known that paraquat exerts its phytotoxic effect by catalyzing the transfer of electrons from photosystem I of chloroplast membranes to molecular oxygen, producing free radicals that cause lipid peroxidation and membrane damage [8]. Plants are known to possess a detoxification system, that allows removal of ROS, consisting of ascorbate and glutathione, as well as enzymatic components, e.g. superoxide dismutase, catalase, ascorbate peroxidase and glutathione reductase [33]. A proposed hypothesis in paraquat resistance is associated with the enhanced activity of antioxidative enzymes functioning in cooperation as a ROS scavenging cycle [34]–[35]. However, the enhanced activity of the enzymes in this cycle could not be detected in most of the paraquat-resistant plants according some earlier observations [11], [14], [36]–[37]. Compared with the transcriptome of untreated goosegrass, most of 53 highly transcribed genes related to ROS scavenging were up-regulated in both of susceptible and resistant biotypes after paraquat treatment. However, the transcripts had no significant differences between RQ and SQ. Therefore, the antioxidant enzyme cycle only provides a temporary protection until other unknown mechanisms in paraquat-resistant plants ensure long-term survival [14].

Polyamines are low molecular weight aliphatic cations that are ubiquitous to all living organisms [38]. Several reports have described that paraquat treatment led to an increase in some polyamines and polyamine feeding also offered high levels of protection against paraquat toxicity [12], [39]–[40]. Pretreatment of radish (*Raphanus sativus* L.) with polyamines (especially 1 mmol/L spermidine) significantly improved their tolerance to subsequent 50 µmol/L paraquat [41]. In the broadleaf weed *Arctotheca calendula*, some polyamines when applied concomitantly with paraquat can reduce the toxicity effects of paraquat. Two polyamines, spermidine and cadaverine, were effective in reducing paraquat translocation in susceptible *A. calendula* inducing these plants to perform more like resistant in terms of translocation [42]. This protective role of polyamines against paraquat stress has been also observed in many plants such as sunflower (*Helianthus annuus* L.) [39], rice (*Oryza sativa* cv. Taichung Native 1) [40], maize (*Zea mays* L. cv. 3377 Pioneer) [16], and some prokaryotes for example *Escherichia coli* [43]–[44]. In our goosegrass transcriptome, among 10 highly transcribed genes related to polyamines, 8 genes were up-regulated after spraying with paraquat in susceptible goosegrass. Polyamines are involved in stress responses as growth regulator. After spraying paraquat, genes related to polyamines were higher compared to the untreated in the susceptible goosegrass. But the susceptible plant resiliency was limited and correspondingly most of genes related to polyamines were lower in sprayed paraquat susceptible plant compared to untreated resistant one. This is indicative of the resistant goosegrass having more polyamines to resist the toxic effects of paraquat. 8 genes were only slightly downregulated in sprayed resistant plant. Lowered levels of five genes were in 0.5, though in gene comp3931_c0_seq1 fold change was 2.45, R0 (RPKM 12.92) were higher than S0 (RPKM 2.20), SQ (RPKM 2.76), and RQ (RPKM 2.36). Gene comp30798_c0_seq2 were upregulated, and the gene expression level was higher, such as R0 (RPKM 59.88) and RQ (RPKM 156.83). Polyamines could protect rice leaves against paraquat toxicity, and paraquat treatment resulted in a higher putrescine and lower spermidine and spermine levels in rice leaves [40]. It suggested that paraquat showed different effects of different polyamines. Thus, our findings confirm that polyamines are involved in paraquat resistance in goosegrass, and the role of different polyamines in paraquat resistance should be further investigated.

Previous reports proposed that some transporters, such as EmrE [45], PotE [46], PrqA, MvrA [47], CAT4 [48], AtPDR 11 [9] and RMV1 [49], are presumed to play a role in the resistance mechanism or to function by carrying paraquat to a metabolically inactive compartment [50]–[51]. In this study, 18 genes corresponded to transmembrane transport, intracellular protein transport and ABC transporters. Most of these genes showed lower level of RPKM in susceptible goosegrass both in untreated and paraquat sprayed plants. 11 of 18 genes related to transport were up-regulated in the treatments between untreated resistant and susceptible goosegrass, while 15 of 18 genes were up-regulated in the treatments of compared resistant and susceptible goosegrass after spraying paraquat. This suggests that some transporters and the transport process they are involved in may play an important function in goosegrass resistance to paraquat.

## Conclusions

The resistant and susceptible biotypes of *E. indica*, with or without paraquat, were used to generate the first large-scale transcriptome sequencing data using Illumina platform. The assembled sequences represented a considerable portion of the transcriptome of this species. The sequence analysis generated 194,716,560 valid reads with an average length of 91.29 bp. *De novo* assembly produced 158,461 transcripts with an average length of 1153.74 bp and 100,742 unigenes with an average length of 712.79 bp. 25,926 unigenes were assigned to 65 GO terms. A total of 13,809 unigenes were assigned to 314 predicted KEGG metabolic pathways, and 12,719 unigenes were categorized into 25 KOG classifications. The polyamine metabolism and transport related genes identified as DEGs provided a functional interpretation of paraquat resistance in goosegrass. Specific functions of these genes in acquired paraquat resistance can be further investigated using the transgenic approach. Collectively, our dataset will serve as a useful resource for further studies on the molecular mechanisms of paraquat resistance and accelerate the discovery of specific paraquat-resistance genes in *E. indica*.

## Materials and Methods

### Plant materials and experimental treatment

A resistant (R) biotype of *E. indica* (goosegrass) was collected from the Teaching and Research Farm (113°40′E, 22°80′N) in Panyu District of Guangzhou, China, where papaya (*Carica papaya* L.) and banana (*Musa nana* Lour.) are cultivated and paraquat is used to control weeds continuously for ∼20 years. The susceptible (S) biotype was collected from the campus of South China Agricultural University (113°36′E, 23°16′N). The paraquat resistant biotype was confirmed prior to performing experiments (Figure 1). Goosegrass seedling cultivation and paraquat treatment were performed as follows: seeds were scarified with sandpaper, sterilized for 10 min in 3% NaClO, washed three times followed by 24 h imbibition in double distilled water, and then germinated in the plastic boxes (22×15.5×7 cm) which contained with a 2∶1∶1 mixture of soil: peat: sand in a growth chamber at 34°C/28°C (day/night) with a 12 h photoperiod at a light intensity of 800±200 µEm^−2^·s^−1^. 14 days after sowing (DAS), seedlings of both S/R biotypes were transplanted into 24 pots (9×7 cm), each containing 6 plants. 21 DAS, both S/R biotypes seedlings at the five leaf stage were sprayed with paraquat (Syngenta, China) of 0.6 kg·ai·ha^−1^ (the recommended rate). The aboveground parts were taken from both untreated seedlings and treated seedlings sprayed with paraquat for 40 min, 60 min and 80 min, respectively. The collected samples were then immediately frozen in liquid nitrogen and stored at −80°C for further experimentation.

Following samples from four different treatments were collected for next-generation sequencing: (1) susceptible goosegrass seedlings without paraquat (S0); (2) susceptible goosegrass seedlings for mixed samples sprayed paraquat 40 min, 60 min and 80 min (SQ); (3) resistant goosegrass seedlings without paraquat (R0); and (4) resistant goosegrass seedlings for mixed samples sprayed paraquat 40 min, 60 min and 80 min (RQ).

### RNA isolation and cDNA library construction

Total RNA was obtained from seedlings using the total RNA purification kit (LC Sciences, Houston, TX, USA) and was further purified using TruSeq RNA LT Sample Prep Kit v2 (Illumina, CA, USA) according to the manufacturer's protocol. Oligo-dT beads were used to yield poly (A+) mRNA from a total RNA pool consisting of equal quantities of total RNA from four sample types of S0, SQ, R0 and RQ. The purified mRNAs were fragmented by using divalent cations under elevated temperatures, and then converted to dsDNA by two rounds of cDNA synthesis using reverse transcriptase and DNA polymerase I. After an end repair process, DNA fragments were ligated with adaptor oligos [24]. The ligated products were amplified using 15 cycles of PCR to generate an RNA-seq library. cDNA sequencing was performed using a Genome Analyzer IIx (Illumina).

### *De novo* assembly and annotation

Raw data generated from Solexa were preprocessed to remove nonsense sequences including (1) adaptor contamination, (2) reads with unknown nucleotides comprising more than 5%, (3) low-quality reads with ambiguous sequence “N”, and (4) very short (35 bp) sequences. Subsequently, *de novo* assembly of the clean reads was performed using assembly program Trinity [52]–[53] which implements a de Bruijn graph algorithm and a stepwise strategy, with the default settings except for the K-mer value (25-mer). After assembly, the longest transcript in each loci (comp*_c*_) was named as “unigene” using Chrysalis Clusters module of Trinity software for subsequent annotation.

For similarity searches, all assembled unigenes were compared with the proteins in the non-redundant (nr) protein database, Swiss-Prot, TrEMBL, CDD, Pfam and KOG databases, respectively, using BLAST with a significance threshold of E-value <10^−5^. Functional categorization by gene ontology (GO) terms was performed by the best BLASTX hits from the nr database using BLAST2GO software according to molecular function, biological process and cellular component ontologies with an E-value threshold of 10^−5^. To further evaluate the integrity of the transcriptome library and the effectiveness of the annotation process, unigenes were subjected to Clusters of Orthologous Groups for Eukaryotic Complete Genomes (KOG) classification. The pathway assignments were carried out by sequence searches against the Kyoto Encyclopedia of Genes and Genomes (KEGG) database and using the BLASTX algorithm with an E-value threshold of 10^−5^.

### Differential gene expression profiling

The expression abundance of each assembled transcript was measured through reads per kilobase per million mapped reads (RPKM) values. All read were mapped onto the non-redundant set of transcripts to quantify the abundance of assembled transcripts. Bowtie was used for read mapping and applied for RPKM based expression measurement. The expressions of each reads between sample pairs (S0 vs SQ, R0 vs RQ, R0 vs S0 and RQ vs RQ) were calculated using the numbers of reads with a specific match. Among the four samples, a minimum of a two-fold difference in log _2_ expression were considered as expression differences.

### Accession for RNA-seq data

The RNA-seq data generated in the study have been uploaded into the NCBI-SRA database under the accession number SRR1181642.

### Ethics statement

We promise that no specific permissions were required for the goosegrass species in the described locations in this manuscript, and the field studies did not involve endangered or protected species.

## References

[1] Holm LG, Plucknett DL, Pancho JV, Herberger JP (1977) The world's worst weeds: University Press. 47–53 p.

[2] Chuah TS, Tan PK, Ismail BS (2013) Effects of adjuvants and soil microbes on the phytotoxic activity of coumarin in combination with p-vanillin on goosegrass (*Eleusine indica* L.) seedling emergence and growth. South African Journal of Botany 84: 128–133.

[3] Lee LJ, Ngim J (2000) A first report of glyphosate-resistant goosegrass (*Eleusine indica* (L) Gaertn) in Malaysia. Pest Management Science 56: 336–339.

[4] Baerson SR, Rodriguez DJ, Tran M, Feng Y, Biest NA, et al (2002) Glyphosate-resistant goosegrass. Identification of a mutation in the target enzyme 5-enolpyruvylshikimate-3-phosphate synthase. Plant Physiology 129: 1265–1275. 12114580 10.1104/pp.001560 PMC166520

[5] Leach GE, Devine MD, Kirkwood RC, Marshall G (1995) Target Enzyme-Based Resistance to Acetyl-Coenzyme A Carboxylase Inhibitors in *Eleusine indica* . Pesticide Biochemistry and Physiology 51: 129–136.

[6] Mudge LC, Gossett BJ, Turphy TR (1984) Resistance of goosegrass (*Eleusine indica*) to dinitroaniline herbicides. Weed science 32: 591–594.

[7] Seng CT, Van Lun L, San CT, SAHID IB (2010) Initial report of glufosinate and paraquat multiple resistance that evolved in a biotype of goosegrass (*Eleusine indica*) in Malaysia. Weed Biol Manag 10: 229–233.

[8] Summers LA (1980) The bipyridinium herbicides: Academic Press Inc. 300–343 p.

[9] Xi J, Xu P, Xiang C-B (2012) Loss of AtPDR11, a plasma membrane-localized ABC transporter, confers paraquat tolerance in *Arabidopsis thaliana* . the Plant Journal 69: 782–791. 22026747 10.1111/j.1365-313X.2011.04830.x

[10] Soar CJ, Karotam J, Preston C, Powles SB (2003) Reduced paraquat translocation in paraquat resistant *Arctotheca calendula* (L.) Levyns is a consequence of the primary resistance mechanism, not the cause. Pesticide Biochemistry and Physiology 76: 91–98.

[11] Yu Q, Cairns A, Powles SB (2004) Paraquat resistance in a population of *Lolium rigidum* . Functional Plant Biology 31: 247–254. 10.1071/FP03234 32688896

[12] Koschnick TJ, Haller WT, Glasgow L (2006) Documentation of landoltia (*Landoltia punctata*) resistance to diquat. Weed Science 54: 615–619.

[13] Hart JJ, Di Tomaso JM (1994) Sequestration and oxygen radical detoxification as mechanisms of paraquat resistance. Weed Science 42: 277–284.

[14] Szigeti Z (2005) Mechanism of paraquat resistance - from the antioxidant enzymes to the transporters. Acta Biologica Szegediensis 49: 177–179.

[15] Hart JJ, DiTomaso JM, Kochian LV (1993) Characterization of paraquat transport in protoplasts from maize (*Zea mays* L.) suspension cells. Plant physiology 103: 963–969. 12231994 10.1104/pp.103.3.963 PMC159070

[16] Hart JJ, DiTomaso JM, Linscott DL, Kochian LV (1992) Transport interactions between paraquat and polyamines in roots of intact maize seedlings. Plant Physiology 99: 1400–1405. 16669051 10.1104/pp.99.4.1400 PMC1080639

[17] DiTomaso JM, Hart JJ, Kochian LV (1993) Compartmentation analysis of paraquat fluxes in maize roots as a means of estimating the rate of vacuolar accumulation and translocation to shoots. Plant physiology 102: 467–472. 12231834 10.1104/pp.102.2.467 PMC158800

[18] Schuster SC (2008) Next-generation sequencing transforms today's biology. Nature Methods 5: 16–18. 18165802 10.1038/nmeth1156

[19] Mardis ER (2008) The impact of next-generation sequencing technology on genetics. Trends in Genetics 24: 133–141. 18262675 10.1016/j.tig.2007.12.007 PMC2680276

[20] Lee J, Noh EK, Choi H-S, Shin SC, Park H, et al (2013) Transcriptome sequencing of the Antarctic vascular plant *Deschampsia antarctica* Desv. under abiotic stress. Planta 237: 823–836. 23135329 10.1007/s00425-012-1797-5

[21] Dang Z-h, Zheng L-l, Wang J, Gao Z, Wu S-b, et al (2013) Transcriptomic profiling of the salt-stress response in the wild recretohalophyte *Reaumuria trigyna* . BMC Genomics 14: 29. 23324106 10.1186/1471-2164-14-29 PMC3562145

[22] Yang X, Yu X-Y, Li Y-F (2013) *De novo* assembly and characterization of the Barnyardgrass (*Echinochloa crus-galli*) transcriptome using next-generation pyrosequencing. PLos One 8: e691687. 10.1371/journal.pone.0069168 PMC3707877 23874903

[23] Zhou Y-J, Gao F, Liu R, Feng J-C, Li H-J (2012) *De novo* sequencing and analysis of root transcriptome using 454 pyrosequencing to discover putative genes associated with drought tolerance in *Ammopiptanthus mongolicus* . BMC Genomics 13: 266. 22721448 10.1186/1471-2164-13-266 PMC3407029

[24] Pruitt KD, Tatusova T, Maglott DR (2005) NCBI Reference Sequence (RefSeq): a curated non-redundant sequence database of genomes, transcripts and proteins. Nucleic Acids Research 33: D501–D504. 15608248 10.1093/nar/gki025 PMC539979

[25] Bairoch A, Apweiler R (2000) The SWISS-PROT protein sequence database and its supplement TrEMBL in 2000. Nucleic Acids Research 28: 45–48. 10592178 10.1093/nar/28.1.45 PMC102476

[26] Boeckmann B, Bairoch A, Apweiler R, Blatter M-C, Estreicher A, et al (2003) The SWISS-PROT protein knowledgebase and its supplement TrEMBL in 2003. Nucleic Acids Research 31: 365–370. 12520024 10.1093/nar/gkg095 PMC165542

[27] Marchler-Bauer A, Lu S, Anderson JB, Chitsaz F, Derbyshire MK, et al (2011) CDD: a Conserved Domain Database for the functional annotation of proteins. Nucleic Acids Research 39: D225–D229. 21109532 10.1093/nar/gkq1189 PMC3013737

[28] Punta M, Coggill PC, Eberhardt RY, Mistry J, Tate J, et al (2012) The Pfam protein families database. Nucleic Acids Research 40: D290–D301. 22127870 10.1093/nar/gkr1065 PMC3245129

[29] Tatusov RL, Fedorova ND, Jackson JD, Jacobs AR, Kiryutin B, et al (2003) The COG database: an updated version includes eukaryotes. BMC Bioinformatics 4: 41. 12969510 10.1186/1471-2105-4-41 PMC222959

[30] Altschul SF, Gish W, Miller W, Myers EW, Lipman DJ (1990) Basic local alignment search tool. Journal of molecular biology 215: 403–410. 2231712 10.1016/S0022-2836(05)80360-2

[31] Bromilow RH (2004) Paraquat and sustainable agriculture. Pest Management Science 60: 340–349. 15119596 10.1002/ps.823

[32] Dori-Bachash M, Shema E, Tirosh I (2011) Coupled Evolution of Transcription and mRNA Degradation. PLos Biology 9: e10011067. 10.1371/journal.pbio.1001106 PMC3139634 21811398

[33] Foyer CH, Descourvieres P, Kunert KJ (1994) Protection against oxygen radicals: An important defence mechanism studied in transgenic plants. Plant Cell and Environment 17: 507–523.

[34] Shaaltiel Y, Gressel J (1986) Multienzyme oxygen radical detoxifying system correlated with paraquat resistance in *Conyza bonariensis* . Pesticide biochemistry and physiology 26: 22–28.

[35] Ye B, Gressel J (2000) Transient, oxidant-induced antioxidant transcript and enzyme levels correlate with greater oxidant-resistance in paraquat-resistant *Conyza bonariensis* . Planta 211: 50–61. 10923703 10.1007/s004250000257

[36] Powles SB, Cornic G (1987) Mechanism of paraquat resistance in *Hordeum glaucum*. I. Studies with isolated organelles and enzymes. Functional Plant Biology 14: 81–89.

[37] Carroll EW, Schwarz OJ, Hickok LG (1988) Biochemical studies of paraquat-tolerant mutants of the fern *Ceratopteris richardii* . Plant Physiology 87: 651–654. 16666201 10.1104/pp.87.3.651 PMC1054814

[38] Kusano T, Berberich T, Tateda C, Takahashi Y (2008) Polyamines: essential factors for growth and survival. Planta 228: 367–381. 18594857 10.1007/s00425-008-0772-7

[39] Benavides MP, Gallego SM, Comba ME, Tomaro ML (2000) Relationship between polyamines and paraquat toxicity in sunflower leaf discs. Plant Growth Regulation 31: 215–224.

[40] Chang CJ, Kao CH (1997) Paraquat toxicity is reduced by polyamines in rice leaves. Plant Growth Regulation 22: 163–168.

[41] Kim H-S, Jin C-D (2006) Polyamines as antioxidant protectors against paraquat damage in radish (*Raphanus sativus* L.) cotyledons. Journal of Plant Biology 49: 237–246.

[42] Soar CJ, Preston C, Karotam J, Powles SB (2004) Polyamines can inhibit paraquat toxicity and translocation in the broadleaf weed *Arctotheca calendula* . Pesticide Biochemistry and Physiology 80: 94–105.

[43] Jung IL, Kim IG (2003) Polyamines reduce paraquat-induced soxS and its regulon expression in *Escherichia coli* . Cell Biology and Toxicology 19: 29–41. 12661985 10.1023/a:1022065614490

[44] Chattopadhyay MK, Tabor CW, Tabor H (2003) Polyamines protect *Escherichia coli* cells from the toxic effect of oxygen. Proceedings of the National Academy of Sciences 100: 2261–2265. 10.1073/pnas.2627990100 PMC151328 12591940

[45] Yerushalmi H, Lebendiker M, Schuldiner S (1995) *Emr*E, an *Escherichia coli* 12-kDa multidrug transporter, exchanges toxic cations and H^+^ and is soluble in organic solvents. Journal of Biological Chemistry 270: 6856–6863. 7896833 10.1074/jbc.270.12.6856

[46] Kashiwagi K, Shibuya S, Tomitori H, Kuraishi A, Igarashi K (1997) Excretion and uptake of putrescine by the PotE protein in *Escherichia coli* . Journal of Biological Chemistry 272: 6318–6323. 9045651 10.1074/jbc.272.10.6318

[47] Nefedova LN, Fantin YS, Zinchenko VV, Babykin MM (2003) The *prq*A and *mvr*A genes encoding drug efflux proteins control resistance to methyl viologen in the *Cyanobacterium Synechocystis* sp. PCC 6803. Russ J Genet 39: 264–268. 12722632

[48] Su YH, Frommer WB, Ludewig U (2004) Molecular and functional characterization of a family of amino acid transporters from *Arabidopsis* . Plant Physiology 136: 3104–3113. 15377779 10.1104/pp.104.045278 PMC523371

[49] Fujita M, Fujita Y, Iuchi S, Yamada K, Kobayashi Y, et al (2012) Natural variation in a polyamine transporter determines paraquat tolerance in *Arabidopsis* . Proceedings of the National Academy of Sciences 109: 6343–6347. 10.1073/pnas.1121406109 PMC3341036 22492932

[50] Jóri B, Soós V, Szegő D, Páldi E, Szigeti Z, et al (2007) Role of transporters in paraquat resistance of horseweed *Conyza canadensis* (L.) Cronq. Pesticide Biochemistry and Physiology 88: 57–65.

[51] Yu Q, Huang S, Powles S (2010) Direct measurement of paraquat in leaf protoplasts indicates vacuolar paraquat sequestration as a resistance mechanism in *Lolium rigidum* . Pesticide Biochemistry And Physiology 98: 104–109.

[52] Grabherr MG, Haas BJ, Yassour M, Levin JZ, Thompson DA, et al (2011) Full-length transcriptome assembly from RNA-Seq data without a reference genome. Nature biotechnology 29: 644–652. 10.1038/nbt.1883 PMC3571712 21572440

[53] Haas BJ, Papanicolaou A, Yassour M, Grabherr M, Blood PD, et al (2013) *De novo* transcript sequence reconstruction from RNA-seq using the Trinity platform for reference generation and analysis. Nat Protoc 8: 1494–1512. 23845962 10.1038/nprot.2013.084 PMC3875132

